# Functional Parameters of ^18^F-FDG PET/CT in Patients with Primary Testicular Diffuse Large B-Cell Lymphoma

**DOI:** 10.1155/2018/8659826

**Published:** 2018-09-27

**Authors:** Jing Yang, Sha Zhu, Fuwen Pang, Miao Xu, Yiting Dong, Jianqi Hao, Xuelei Ma

**Affiliations:** ^1^State Key Laboratory of Biotherapy and Cancer Center, West China Hospital, Sichuan University and Collaborative Innovation Center for Biotherapy, Chengdu, China; ^2^West China School of Medicine, West China Hospital, Sichuan University, Chengdu 610041, China; ^3^Department of Pathology, West China Hospital, Sichuan University, Chengdu, China

## Abstract

Fluorine-18 fluorodeoxyglucose (^18^F-FDG) positron-emission tomography/computed tomography (PET/CT), a hybrid imaging technique that simultaneously provides functional and anatomical information, has been reported to be useful in lymphoma. The present study was to evaluate the functional parameters of ^18^F-FDG PET/CT in patients with testicular diffuse large B-cell lymphoma (DLBCL). We retrospectively reviewed medical records of 5095 patients with lymphoma who treated at West China Hospital between March 2003 and January 2017, and selected patients with ^18^F-FDG PET/CT findings and subsequently biopsy confirmed the invasion of testis with DLBCL. Maximum standardized uptake values (SUV_max_), peak standardized uptake values (SUV_peak_), metabolic tumor volume (MTV), and total lesion glycolysis (TLG) of the patients were measured. We evaluated the characteristics of ^18^F-FDG PET/CT in this population. Six patients ranged in age from 37 to 73 years (median age, 58 years) were included in the analysis. The mean SUV_max_ was 11.09 and varied between 7.20 and 19.75; mean SUV_peak_ was 9.56 and ranged between 6.79 and 14.39. In addition, mean MTV 42% was 18.4 and varied between 1.3 and 61.6; mean MTV 2.5 was 34.7 and varied significantly between 1.6 and 141.9. With regard to TLG, mean TLG 42% was 168.906 and ranged from 7.514 to 687.004, while mean TLG 2.5 was 253.972 and ranged from 8.400 to 1127.802. In conclusion, ^18^F-FDG PET/CT scan is a useful tool in patients with testicular DLBCL. SUV, MTV, and TLG may vary a lot in different patients. SUV_max_ of testicular DLBCL lesion is relatively higher than that of normal testis. Also, we provided a set of MTV and TLG data and firstly showed their significant correlation with overall survival, which indicated a potential prognostic value of MTV and TLG. However, studies with larger population are needed to confirm these findings.

## 1. Introduction

Testicular lymphoma is a rare but aggressive form of extranodal lymphoma, accounting for 3–9% of testicular cancers and 1-2% of non-Hodgkin's lymphomas [1, 2]. In spite of the low overall incidence, testicular lymphoma is the most common testicular malignancy in men over 60 [2]. Testicular diffuse large B-cell lymphoma (DLBCL) is the most common histological subtype, accounting for about 80% to 98% of all cases [3]. Although radical inguinal orchiectomy is recommended in view of histological evaluation, invasiveness often hinders its wider adoption [4]. Other diagnostic methods include testicular ultrasound, computed tomography (CT), routine blood test, lactate dehydrogenase, bone marrow biopsy, and lumbar puncture [5].

Fluorine-18 fluorodeoxyglucose (^18^F-FDG) positron-emission tomography/computed tomography (PET/CT) is a hybrid imaging technique that simultaneously provides functional and anatomical information. ^18^F-FDG PET/CT is important in biomedical research and clinical diagnostics, and its application in lymphoma has already been reported [6, 7]. There are several parameters being repeatedly discussed in recent studies, including maximum standardized uptake values (SUV_max_), peak standardized uptake values (SUV_peak_), metabolic tumor volume (MTV), and total lesion glycolysis (TLG), since they are believed to play important roles in the diagnosis and prognosis of patients with lymphoma [8–10]. Therefore, the National Comprehensive Cancer Network (NCCN) guidelines recommend the use of ^18^F-FDG PET/CT for staging, response evaluation, and prognosis of lymphoma.

However, the role of ^18^F-FDG PET/CT among patients with testicular DLBCL has still not been well established. In this study, we reported 6 patients with testicular DLBCL who had performed ^18^F-FDG PET/CT scan and discussed the role of ^18^F-FDG PET/CT in this population at the same time.

## 2. Materials and Methods

### 2.1. Patients

We retrospectively reviewed medical records of 5095 patients with lymphoma. All patients were treated at West China Hospital between March 2003 and January 2015. Inclusion criteria were as follows: (1) ^18^F-FDG PET/CT findings before receiving orchiectomy were present and (2) subsequent biopsy confirmed the invasion of testis with DLBCL. Patients' characteristics including the histological type, Ann Arbor stage, International Prognostic Index (IPI) score, NCCN IPI score, ECOG performance status, B symptom, metastatic sites, and treatment were extracted. This study was approved by the Ethics Administration Office of West China Hospital, Sichuan University.

### 2.2. ^18^F-FDG PET/CT Imaging

**Figure 1:**
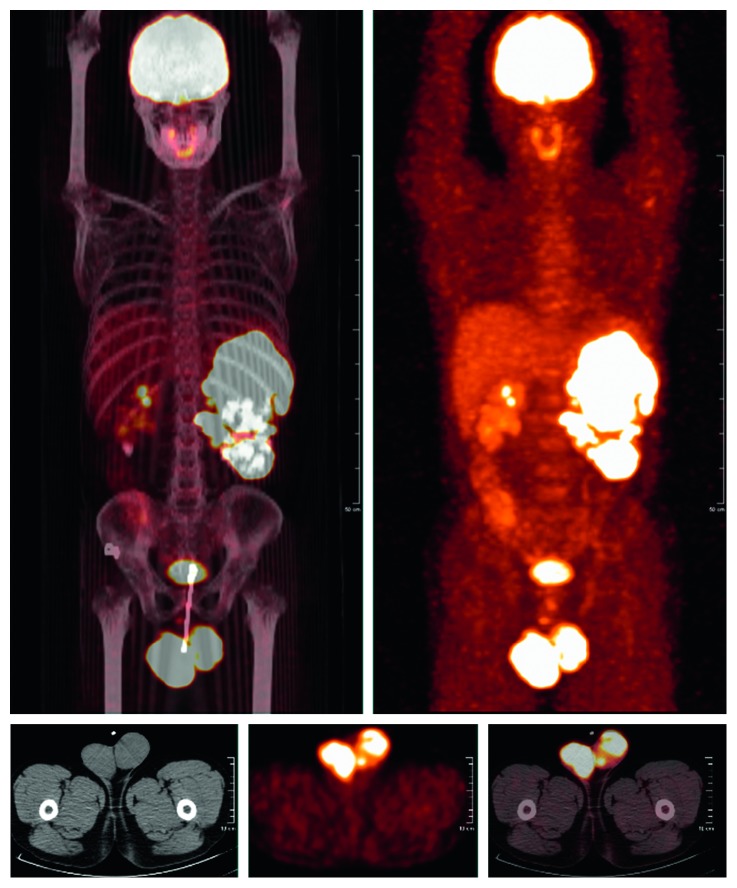
A 37-year-old patient was diagnosed with diffuse large B-cell lymphoma that involved with bilateral testes. The PET/CT showed asymmetrical increased uptake in the bilateral testes. He received orchiectomy, prophylactic intrathecal injection, and 6 cycles of chemotherapy with rituximab-etoposide, prednisone, oncovin (vincristine), cyclophosphamide, and hydroxydaunorubicin (doxorubicin) (R-DA-EPOCH). The duration from the time of diagnosis to the date when radiological findings suggested suspected pancreatic involvement was 9.3 months. The patient was alive till October 30, 2017, after a follow-up of 26 months.

**Figure 2:**
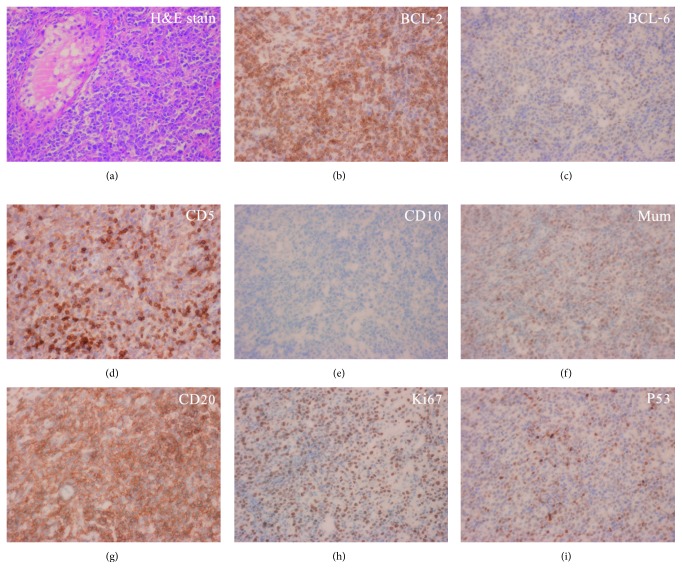
Histopathological (H&E stain (×400)) and immunohistochemical (×400) findings of the testicular lymphoma biopsy specimen of the 37-year-old patient: BCL-2 (+), BCL-6 (+), CD5 (+), CD10 (−), CD20 (+), Mum (+), Ki-67/MIB-1 (+, 80%), and P53 (+).

Standard whole-body ^18^F-FDG PET/CT was performed using a Gemini GXL PET/CT scanner (Philips, Amsterdam, The Netherlands). Fasting for at least 6 hours was required before the examination, and the blood glucose level was measured immediately before the administration of ^18^F-FDG. The PET/CT scan would be rescheduled if the blood glucose level was >150 mg/dL. Approximately 5 MBq of ^18^F-FDG per kilogram of body weight was administered intravenously, and the patients rested in a quiet, dark environment for approximately 60 minutes before scanning. After initial low-dose CT (40 mA, 120 kVp), emission images were obtained from the top of the skull to the middle of the thigh, with acquisition times of 2 minutes per bed position in the three-dimensional mode. The PET images were reconstructed iteratively with CT-based attenuation correction (Figures 1 and 2).

### 2.3. Image Analysis

The image analysis was performed using Compass Viewer software. Circular regions of interest (ROIs) were manually drawn on axial, coronal, or sagittal coregistered PET/CT slices. Within the selected ROI, SUV_max_, mean standardized uptake values (SUV_mean_), SUV_peak_, MTV, and TLG were measured. SUV_max_ were calculated using the following formula: mean ROI activity (MBq/g)/(injected dose (MBq)/body weight (g)). SUV_peak_ was defined as the mean of SUV_max_ and its 10 neighbors (roughly corresponding to a 0.5 cm ROI). MTV and TLG could be measured by a fixed background SUV cut-off or a fixed percentage of the SUV_max_. In this study, we calculated SUV_mean_ and MTV based on a fixed threshold of 42% of SUV_max_ (SUV_mean_ 42%, MTV 42%) or based on a fixed background SUV cut-off of 2.5 (SUV_mean_ 2.5, MTV 2.5). TLG was defined as the MTV multiplied with the SUV_mean_ (TLG 42%, TLG 2.5).

### 2.4. Statistical Analysis

Correlation analysis between the functional parameters of ^18^F-FDG PET/CT and overall survival (OS) was conducted, and Spearman's rank coefficients were used to assess the relationship between the functional parameters and outcomes of the patients. Statistical analyses were performed using the SPSS version 22.0 (IBM Corporation, Armonk, NY, USA) at a significance level of *p* < 0.05.

## 3. Results

A total of 34 patients with testicular lymphoma were selected from this population. Eighteen of them had ^18^F-FDG PET/CT findings while only 6 had preoperative images. As a result, 6 patients ranging from 37 to 73 years old (median age, 58) were included in the analysis. Patients' characteristics including the histological type, Ann Arbor stage, IPI score, NCCN IPI score, ECOG performance status, B symptom, metastatic sites, and treatment are described in Table 1. All patients had histopathological confirmation of DLBCL. Five (83.3%) out of 6 patients were classified clinically as stage IVB, and 1 (16.7%) as stage IEA according to the Ann Arbor classification. The IPI score of patients were calculated, and the results revealed that 5 patients (83.3%) had a score of 3 while 1 patient (16.7%) had a score of 1. In addition, 1 (16.7%) patient had an ECOG performance status of 1, while 5 patients (83.3%) had an ECOG performance status of 0. Of the 6 patients, 3 (50%) had tumor located on the left side and 1 (16.3%) on the right side, whereas 2 (33.3%) on the bilateral sides. Besides testicular disease, 5 of the patients were identified to have lymph nodes or other distant metastases. All the patients have received treatment, of whom 6 (100%) had orchiectomy and chemotherapy, 2 patients (33.3%) had local radiotherapy, and 4 (66.7%) received prophylactic intrathecal injection in addition to their systemic chemotherapy. Adjunct laboratory and immunohistochemical results of the patients, such as Ki-67, *β*_2_ microglobulin, and LDH, are also shown in Table 1.

Within the selected ROI, SUV_max_, SUV_mean_, SUV_peak_, MTV, and TLG were measured. The mean SUV_max_ was 11.09 and varied between 7.20 and 19.75; mean SUV_peak_ was 9.56 and ranged between 6.79 and 14.39. In addition, mean MTV 42% was 18.4 mL and varied between 1.3 mL and 61.6 mL; mean MTV 2.5 was 34.7 mL and varied significantly between 1.6 mL and 141.9 mL. With regard to TLG, mean TLG 42% was 168.906 and ranged from 7.514 to 687.004, while mean TLG 2.5 was 253.972 and ranged from 8.400 to 1127.802 (Table 2).

The result of correlation analysis between functional parameters and survival time indicated that SUV_max_ and SUV_peak_ were not significantly associated with OS of the patients. However, MTV 42%, MTV 2.5, TLG 42%, and TLG 2.5 were revealed to be significantly correlated with OS of the patients, with Spearman's rank coefficients of 0.812 and *p*=0.04982 (Table 3).

## 4. Discussion

^18^F-FDG PET/CT is performed in combination with ^18^FDG PET and CT scanners.^18^F-FDG PET/CT has been reported to be a very useful tool with high sensitivity and specificity rates in evaluating most lymphoma subtypes, providing both metabolic and morphologic features of diseases [11]. Compared with contrast-enhanced CT (CECT), PET/CT shows a higher diagnostic value with sensitivity of 97% and specificity of 100%, especially for normal-sized lymph nodes and extranodal involvement [12–14]. Moreover, with the supplement of other examinations, ^18^F-FDG PET/CT can not only make accurate diagnosis but also assess the treatment response as well as predict the outcomes [15–18]. However, as far as we know, the application of PET/CT in testicular DLBCL patients has not been well studied. In this study, we firstly focused on the use of ^18^F-FDG PET/CT in the prognosis and staging of patients with testicular DLBCL and reported their SUV_max_, SUV_mean_, SUV_peak_, MTV, and TLG.

Because of its aggressive clinical biological behavior, patients with testicular lymphoma usually present a poor prognosis. Timely and accurate diagnosis of testicular lymphoma is vital since early diagnosis was reported to be associated with better outcomes [19]. Imaging modalities that may be helpful in diagnosis include ultrasonography, magnetic resonance imaging, and CT, while unfortunately none of these methods shows satisfying specificity [3]. Fine-needle aspiration, testicular biopsy, and orchiectomy have been used for pathological diagnosis of testicular lymphoma. Nevertheless, these pathological diagnostic process may do harm to the testes' physiological functions as well as patients' mental health [3]. PET/CT is now widely used in the diagnosis and initial staging of high-grade lymphoma [20]. In this study, we also demonstrated the value of PET/CT in diagnosis and staging among patients with testicular DLBCL.

SUV_max_, the most widely used parameter, is a reproducible measurement for disease evaluation in a quantitative way [21]. Previous studies have reported that the normal level of FDG uptake in the testis is relatively high and symmetrical in pattern and declines slightly with age [22]. A study involving 203 men has demonstrated that the normal SUV range from 1.23 to 3.85 with a mean value of 2.44 [23]. In addition, previous study including 53 patients has reported that a SUV_max_ of 3.75 is the optimal cut-off value for differentiating between benign and malignant testicular diseases [24]. As for the testicular lesions of our population, the mean SUV_max_ was 11.09, with a range of 7.20 to 19.75. SUV_max_ of all our patients were larger than 3.75. The results of this study revealed a high FDG uptake in testicular DLBCL patients; therefore, abnormal uptake of FDG in testis warranted further analysis. In addition, the value of SUV_peak_ was also shown in this study. However, to the best of our acknowledgement, no previous studies have reported these indexes of testicular DLBCL patients.

MTV and TLG can be measured by a fixed background SUV cut-off or a fixed percentage of the SUV_max_ [25–27]. Both MTV and TLG have been proposed to assess the burden of metabolically active tumors and are assumed to be reliable indicators of the tumor bulk [28]. In this study, we calculated MTV 42%, MTV 2.5, TLG 42%, and TLG 2.5 of each patient. The mean MTV 42% was 18.4 mL while the mean MTV 2.5 was 34.7 mL; meanwhile, both of them showed an apparent change. MTV of tumor burden has been recently found to be a useful prognostic factor in lymphoma [29]. TLG, which combined the volumetric and metabolic information of ^18^F-FDG PET, was also calculated in this study. Elevated TLG has also been shown to be associated with poor survival in various types of cancer, but its prognostic value in testicular lymphoma has not been well established [30]. As a result, we demonstrated that MTV and TLG may greatly differ between different patients. The values of MTV and TLG in neither normal testes nor testicular lymphoma have been investigated; thus, further studies are expected. To the best of our knowledge, we assessed the correlation between functional parameters of ^18^F-FDG PET/CT and survival of patients with primary testicular DLBCL for the first time. MTV and TLG were shown to be correlated with survival with statistical significance, which indicated a potential prognostic value of MTV and TLG. However, further studies are needed to confirm these results.

The current study has several limitations. First, this is a retrospective analysis. Second, the number of patients is small. Although we identified 18 testicular DLBCL with PET/CT scan, 12 of them had undergone orchiectomy before PET/CT examination. As a result, testicular disease could not be identified in the scan. Third, population from a single center also limits the conclusions of our study. Thus, further prospective randomized studies using multicenter data are required to confirm our findings. The strength of this study includes that histological confirmation of testicular DLBCL was obtained in all the patients, and patients' data were complete.

## 5. Conclusions

In conclusion, PET/CT scan has the potential in evaluating patients with testicular DLBCL. SUV, MTV, and TLG may vary a lot in different patients. SUV_max_ of testicular DLBCL lesion is relative higher than that of normal testis. Also, we provided a set of MTV and TLG data and firstly showed their significant correlation with OS, which indicated a potential prognostic value of MTV and TLG. However, studies with a larger population are needed to confirm these findings.

## Figures and Tables

**Table 1:** Baseline characteristics of the patients.

No	Age	Ann Arbor stage	IPI score	ECOG performance status	Site	Nodal involvement	Exnodal involvement	Ki-67	*β*_2_ microglobulin (mg/L)	LDH (IU/L)	Treatment
1	37	IVB	3	0	Bilateral	Para-aortic lymph node	Bilateral kidney, perirenal region, and spleen	80%	2.33	237	Orchiectomy, CT, and prophylactic intrathecal injection
2	73	IVB	3	0	Right	Abdominal lymph node	Lung and nasopharyngeal wall	40%	2.63	177	Orchiectomy, CT, RT, and prophylactic intrathecal injection
3	57	IVB	3	1	Left	Neck lymph node	Maxillary sinus, maxillary bone, orbital cavity, temporalis, multiple subcutaneous tissue, and bone of trunk	60%	2.82	301	Orchiectomy and CT
4	58	IEA	1	0	Left	—	—	N/A	N/A	223	Orchiectomy, CT, and RT
5	73	IVA	3	0	Bilateral	Cervical lymph nodes and hilar lymph node	Skin	50%	2.19	246	Orchiectomy, CT, and prophylactic intrathecal injection
6	58	IVB	3	0	Left	Multiple lymph nodes	Kidney, adrenal gland, and spermatic cord	90%	NA	367	Orchiectomy and CT + prophylactic intrathecal injection

IPI, International Prognostic Index; NCCN IPI, National Comprehensive Cancer Network International Prognostic Index; ECOG performance status, Eastern Cooperative Oncology Group performance status; LDH, lactic dehydrogenase; CT, chemotherapy; RT, radiotherapy; N/A, not applicable.

**Table 2:** SUV, MTV, TLG, and survival of the patients.

No.	SUV_max_	SUV_peak_	MTV 42%	MTV 2.5	TLG 42%	TLG 2.5	Overall survival (months)	Outcomes
1	19.75	14.39	61.6	141.9	687.004	1127.802	26	Alive
2	11.30	9.52	13.1	16.9	91.045	105.794	54	Death
3	7.98	6.99	3.8	4.8	20.786	23.808	17	Alive
4	11.90	11.56	20.7	31.7	168.912	209.537	22	Death
5	7.20	6.79	9.7	11.2	44.175	48.49	18	Alive
6	8.40	8.11	1.3	1.6	7.514	8.400	17	Alive

SUV_max_, maximum standardized uptake values; SUV_peak_, peak standardized uptake values; MTV, metabolic tumor volume; TLG, total lesion glycolysis.

**Table 3:** Spearman rank correlation for functional parameters and overall survival.

	SUV_max_	SUV_peak_	MTV 42%	MTV 2.5	TLG 42%	TLG 2.5
Spearman rank correlation coefficient	0.638	0.638	0.812	0.812	0.812	0.812
*p* value	0.1731	0.1731	0.0498	0.0498	0.0498	0.0498

SUV_max_, maximum standardized uptake values; SUV_peak_, peak standardized uptake values; MTV, metabolic tumor volume; TLG, total lesion glycolysis.

## Data Availability

The data used to support the findings of this study are available from the corresponding author upon request.

## Abbreviations

DLBCL: Diffuse large B-cell lymphoma
CT: Computed tomography
^18^F-FDG: Fluorine-18 fluorodeoxyglucose
PET/CT: Positron-emission tomography/computed tomography
SUV_max:_: Maximum standardized uptake values
SUV_peak:_: Peak standardized uptake values
SUV_mean:_: Mean standardized uptake values
MTV: Metabolic tumor volume
TLG: Total lesion glycolysis
NCCN: National Comprehensive Cancer Network
IPI: International Prognostic Index
ROI: regions of interest
OS: Overall survival.
